# Signes cliniques rencontrés chez l'enfant malnutri dans un milieu minier: cas de la ville de Lubumbashi et ses environs

**DOI:** 10.11604/pamj.2016.24.67.9146

**Published:** 2016-05-17

**Authors:** Aimée Mudekereza Musimwa, Gray Wakamb Kanteng, Hermann Tamubango Kitoko, Oscar Numbi Luboya

**Affiliations:** 1 Département de Pédiatrie, Faculté de Médecine Université de Lubumbashi, Lubumbashi, République Démocratique du Congo

**Keywords:** Malnutrition, enfant, signes cliniques, milieu minier, Lubumbashi, Malnutrition, child, clinical, mining environment, Lubumbashi

## Abstract

**Introduction:**

La malnutrition reste à ce jour un problème majeur de santé publique, notamment dans les pays en voie de développement. Cette étude a eu pour objectif de déterminer les signes cliniques observés chez l'enfant mal nourri, admis dans une unité de prise en charge.

**Méthodes:**

Il s'agit d'une étude descriptive transversale, effectuée de juillet 2013 à décembre 2014. 311 cas ont été colligés (182 malnutris et 129 biens nourris), par échantillonnage exhaustif, avec un dépistage actif des enfants malnutris et biens nourris. Le diagnostic est fait cliniquement associé à l'anthropométrie.

**Résultats:**

Les signes les plus enregistrés chez les enfants malnutris étaient dominés par la toux ou pneumopathie dans 42,50%, la gastroentérite dans 38,55%, les lésions dermatologiques ont présenté 22,91% de cas, la fièvre dans 22,35% de cas, 19,0% des enfants ont présenté les œdèmes, 8,38% d'enfants ont présenté la pâleur, enfin hépatomégalie et splénomégalie sont les signes les moins fréquents avec respectivement 1,68% et 2,89%. Tandis que les enfants en bonne état nutritionnel ont présenté plus la splénomégalie et la fièvre qui seraient liés au paludisme.

**Conclusion:**

Les enfants malnutris vivant dans un milieu minier présentent une symptomatologie qui ne pas différents de autres enfants mal nourris à l'exception de l'hépatomégalie et splénomégalie qui sont très rare chez nos mal nourris.

## Abstract (en)

**Introduction:**

Malnutrition remains to this day a major public health problem, particularly in developing countries. This study aimed to determine the clinical signs observed in malnourished children admitted to a care unit.

**Methods:**

This is a descriptive cross-sectional study, conducted from July 2013 to December 2014. Our study included 311 cases (182 malnourished children and 129 well-nourished children), based on exhaustive sampling, with an active screening of malnourished and well-nourished children. The diagnosis was made clinically and was associated with anthropometry.

**Results:**

The main collected symptoms in malnourished children were: cough or pneumonia in 42.50%, gastroenteritis in 38.55%, skin lesions in 22.91% of cases, fever in 22.35% of cases, edema in 19.0% of children, pallor in 8.38% of children; finally splenomegaly and hepatomegaly were the less common symptoms (1.68% and 2.89% respectively). Well-nourished children, instead, showed splenomegaly and fever associated with malaria.

**Conclusion:**

Malnourished children living around a mining area don't differ in symptomatology from the other malnourished children, except for hepatomegaly and splenomegaly which are very rare in our malnourished children.

## Introduction

La malnutrition est un problème de santé publique chez les enfants et en Afrique sub-saharienne. L'exposition aux métaux toxique et la carence en oligo-éléments constituent un véritable problème de santé publique en raison de la qualité nutritionnelle insuffisante de nourriture et du stockage des métaux lourds. Les carences en micronutriments provoquent des interactions complexes qui conduisent au cercle vicieux de la malnutrition et des infections [1]. Les taux de malnutrition infantile en RD Congo restent très élevés dans les provinces qui dépendent de l'industrie minière et comparable au niveau observé dans les provinces de l'Est en proie aux conflits [2]. Dans les régions minières, une exposition prolongée aux facteurs nuisibles environnementaux ou aux déchets miniers qui entraîneraient un retard dans la croissance et endommage le développement du cerveau des enfants. Le cerveau d'un enfant est plus vulnérable aux dommages causés par des agents toxiques [3]. Les effets des produits chimiques environnementaux sur la santé des enfants ont été largement signalés; la majorité se concentrant sur les effets nocifs sur le système nerveux central (SNC) [4]. En outre, le comportement des enfants de porter la main à la bouche ainsi que de jouer près du sol augmente également leur probabilité d'exposition [4, 5]. Plusieurs études ont démontré récemment, les effets indésirables sur la santé des enfants liés à l'exposition à des métaux, où les principales conséquences ont été les déficits de l'enseignement, de l'attention et une atteinte rénale [6–8]. Selon la banque mondiale et l'OMS, la prévalence globale de la malnutrition en RDC est passée de 24,2% à 23,4% de 2010 à 2013[9]. La province du Katanga région minière, se retrouve en deuxième position après la province du Maniema où la malnutrition et la mortalité infantile sont les plus élevées en RD Congo [10]. A Lubumbashi, il a été observé un retard de croissance de 33,5% et de 3,8% l'amaigrissement [11]. La réalisation de cette étude se justifie avant tout par le besoin de connaitre les signes cliniques que présenteraient les enfants malnutris dans une région minière. Les taux plasmatiques en protéines et en éléments traces métalliques sont logiquement influencés par les apports alimentaires au niveau des ménages et par l'exposition aux polluants et toxiques divers engendrés par la production, le stockage et le transport des minerais à travers les milieux d'habitations. A Lubumbashi, depuis l'effervescence des industries minières, les études fiables portant sur la clinique de l'enfant malnutri sont rares. D'où la question de savoir quelle est la clinique présentée par l'enfant malnutri âgé de 0 à 5 ans à Lubumbashi et ses environs? L'objectif principal de cette étude a été de déterminer la clinique des enfants malnutris admis dans une unité de prise en charge dans la ville de Lubumbashi et ses environs.

## Méthodes

Il s'agit d'une étude descriptive transversale couvrant la période du 01 juillet 2013 au 31 décembre 2014, effectuée dans la zone urbaine et péri urbaine de Lubumbashi, au sud-est de la République Démocratique du Congo. Les sites retenus ont été l'hôpital Général de Référence Jason Sendwe, l'hôpital Général de Référence Kisanga, l'hôpital Militaire Camp Vangu, l'hôpital de Référence Mamba 2 et dans le village Kawama situé à 30 km de la ville de Lubumbashi sur la route de Likasi. Notre étude a porté sur un échantillonnage de 311 enfants âgés de 06 à 59 mois, soit 182 malnutris nouvellement admis au centre de réhabilitation ou de prise en charge pour prise en charge de malnutrition aigüe sévère et n'ayant pas encore reçu de traitement de prise en charge de la malnutrition, et 129 en bonne état nutritionnel recrutés dans différents dispensaires de pédiatrie de hôpitaux précités. Le diagnostic de malnutrition a été défini selon les critères de l'Organisation Mondiale de la Santé (OMS, 2006) par: l'indice poids-âge inférieur à -2 écarts types et périmètre brachial inférieur à 115 mm ou œdèmes bilatéraux et signes cliniques de malnutrition. Les variables étudiées ont été: l’âge, le poids (P), taille (T); ces trois éléments permettant de rechercher les différents z-scores. Un prélèvement de sang veineux a été effectué chez chacun d'entre eux. Les éléments cliniques suivants ont été étudiés: la toux, la gastroentérite, les dermatoses, la fièvre, la pâleur, les œdèmes et l'hépato-splénomégalie. Les données ont été saisies et analysées sur Epi info version 3.5.2. L'analyse statistique a porté sur des comparaisons unies variées. Les fréquences, les moyennes, les médianes, les pourcentages ainsi que les déviations standards ont étés générés à intervalles de confiance (IC) à 95%. Le protocole du travail a été soumis et approuvé au Département de Pédiatrie des Cliniques Universitaires de Lubumbashi. Nos prélèvements ont été effectués sous le consentement verbal libre et éclairé des parents de chaque enfant, après une explication brève du but de notre étude.

## Résultats

### Données sociodémographiques

**Figure 1:**
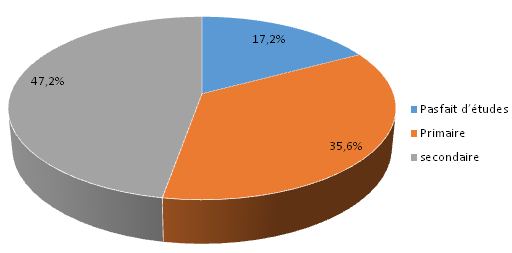
Répartition selon le niveau d’étude de la mère

Il ressort (Tableau 1) que l’âge médian de nos patients est de 24 mois par contre il est de 36 mois pour le groupe témoins avec les extrêmes pour toutes les deux catégories de 6 à 59 mois. Il ressort (Tableau 2) que 100 enfants malnutris (soit 55,87%) étaient du sexe féminin contre 79 enfants malnutris (soit 44,13%) qui étaient du sexe masculin soit un sexe ratio de 0,7 chez les enfants malnutris. De ce même tableau, il ressort que 74 enfants biens nourris (soit 57,36%) étaient du sexe masculin contre 55 enfants témoins (soit 42, 64%) qui étaient du sexe féminin soit un sexe ratio de 1,34. Les résultats de la Figure 1 montrent que 47,2% des mères avaient un niveau d’études secondaires, contre 17,2% qui représentés les mères sans études faites.

**Tableau 1:** Répartition de cas en fonction l’âge

Catégorie	Effectif	Age médian (en mois)	Minimum	Maximum
malnutris	179	24	6	59
Biens nourris	128	36	6	59

**Tableau 2:** Répartition des cas en fonction du sexe

Sexe	Catégorie		Total
	malnutris	Biens nourris	
F	100 (55,87%)	55 (42,64%)	155(50,32%)
M	79(44,13%)	74(57,36%)	153(49,68%)
Total	179 (58,12%)	129(41,88%)	308(100%)

### Données nutritionnelles

A l'examen du Tableau 3, il ressort que seul 51% d'enfants entre 23 et 59 mois bénéficiaient d'une alimentation faite de plats familiaux contre 0,6% de 0 à 3 mois et 0,6% de 11 à 23 mois qui étaient exclusivement nourris au lait maternel.

**Tableau 3:** Répartition selon l’âge et le mode d'alimentation chez les malnutris

Age (mois)	Lait maternel seul		Mixte		Plat familial	
	Effectif	%	Effectif	%	Effectif	%
[0–6]	1	0,6	10	5,6	0	0
[6–12]	4	2,2	13	7,2	5	2,8
[12–24]	1	0,6	12	6,7	26	14,4
[24–59]	0	0	16	8,9	94	51

### Données anthropométriques

A l'analyse du Tableau 4, 23,3% des enfants présentaient une légère malnutrition, 51,1% des enfants avaient une malnutrition modérée tandis que 25,6% d'entre eux étaient sévèrement malnutris. (Selon classification de Kanawati; 1970). Par rapport au paramètre poids pour âge, 37,2% d'enfants étaient en insuffisance pondérale modérée contre 32,8% d'entre eux qui présentaient une insuffisance pondérale sévère. Concernant le paramètre taille pour âge 85% d'enfants souffraient de malnutrition chronique, soit 22,2% de forme modéré et 62,8% de forme sévère et pour le poids pour taille; 15,5% d'enfants souffraient de malnutrition aiguë; soit 11,1% d'enfants qui présentaient une malnutrition aiguë modérée et 4,4% d'entre eux souffrait de malnutrition aiguë sévère. Par rapport à la provenance, au moins de 47% patients résidaient en zone rurale à savoir le village Kawama.

**Tableau 4:** Répartition patients selon l'anthropométrie

Paramètres	Fréquence (n = 182)	Pourcentage
**Z-score Poids/Age**		
**< -3**	61	32,8
**[- 3 à -2]**	67	37,2
**> - 2**	54	30
**Z-score Taille/Age**		
**< - 3**	115	62,8%
**[-3 à - 2]**	40	22,2%
**> -2**	27	15%
**Z-score Poids/Taille**		
**< - 3**	10	4,4
**[-3 à - 2]**	20	11,1
**>-2**	152	84,5
**PB/PC**		
**< 0,25**	48	25,6%
**[0,25 - 0,28]**	92	51,1%
**]0, 28 -0, 30]**	42	23,3%

### Les critères cliniques

**Figure 2:**
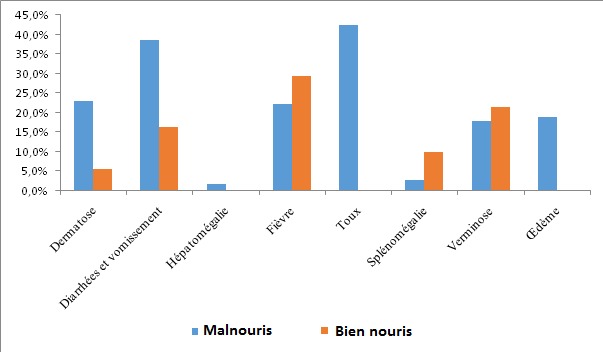
Les signes cliniques les plus rencontrés

La Figure 2 montre que les signes les plus enregistrés chez les enfants malnutris étaient dominés par la toux ou pneumopathie dans 42,50%, la gastroentérite dans 38,55%, les lésions dermatologiques ont présenté 22,91% de cas, la fièvre dans 22,35% de cas, 19,0% des enfants ont présenté les œdèmes, 8,38% d'enfants ont présenté la pâleur, enfin Hépatomégalie et splénomégalie sont les signes les moins fréquent avec respectivement 1,68% et 2,89%. Tandis que les témoins ou les enfants en bonne état nutritionnel ont présenté plus la splénomégalie et la fièvre; qui seraient liés au paludisme.

## Discussion

La répartition des cas en fonction de l’âge (Tableau 1) montre que l’âge médian de nos patients est de 24 mois; par contre il est de 36 mois pour le groupe bien nourris avec les extrêmes pour toutes les deux catégories de 6 à 59 mois. Nous constatons que les enfants en bonne état nutritionnel étaient plus âgés que les malnutris. Cette médiane de 24 mois se retrouve dans d'autres études menées par Sinnaeve et al 2006; Ouattara et al 2007 au Bénin [12, 13] et s'expliquerait probablement par le fait que cette classe d’âge correspond à la période de sevrage des enfants et de passage à l'alimentation familiale, qui la plupart du temps n'est pas réalisée dans les conditions idéales. Il intervient le plus souvent brutalement lorsque l'enfant atteint l’âge d’être sevré ou, au cours d'une maladie de l'enfant ou en raison d'une nouvelle grossesse; ce qui rend ce cap difficile à franchir par l'enfant entrainant ainsi la rupture de l’équilibre nutritionnel [14]. Pour Barry Boubacar et al 2009 [15], la tranche d’âge de 12 à 23 mois était la plus représentée, suivie de la tranche d’âge de 24 à 59 mois. La classe la moins touchée par toutes les formes de malnutrition est celle de 0 à 6 mois; Ce phénomène s'expliquerait par la protection qu'offre l'allaitement maternel pour cette tranche d’âge. Nos enfants malnutris sont légèrement âgés que ceux présentés dans l’étude de Mpoy et al 2014 [16] qui présente une médiane de 23 mois.

La répartition des enfants en fonction du sexe (Tableau 2), montre que 100 enfants malnutris (soit 55,87%) étaient du sexe féminin contre 79 enfants malnutris (soit 44,13%) qui étaient du sexe masculin soit un sexe ratio de 0,7 chez les enfants malnutris. De ce même tableau, il ressort que 74 enfants biens nourris (soit 57,36%) étaient du sexe masculin contre 55 enfants (soit 42, 64%) qui étaient du sexe féminin soit un sexe ratio de 1,34. Ceci veut dire que l'on a eu plus des malnutris dans le sexe féminin. Concernant les malnutris, nos résultats sont identiques à ceux Ilboudo N 2004 [17] qui a mené une étude au Burkina Faso sur les déterminants de la malnutrition des enfants et qui soutient que le sexe de l'enfant est un déterminant important pour son état de santé et que la malnutrition chronique est plus importante chez les filles que chez les garçons. Hayfa Grira 2007 [18] qui indique que la malnutrition est plus importante chez les filles que chez les garçons. Contrairement à nous, Boubacar 2009 et Kabirou 2002 [15, 19] rapportent quant à eux, une plus grande atteinte masculine. Au Niger le Rapport d'enquête nationale de nutrition montre que la malnutrition aiguë touche davantage plus les enfants de sexe masculin que leurs sœurs du même âge avec des taux respectifs de 15,0% et 11,6%. Nous pensons donc dans notre étude que, la prédominance féminine pourrait être expliquée par le fait que durant notre étude nous avons retenu plus de filles sur l'effectif total.

### La provenance et niveau socio-économique

47,8% des 180 enfants de notre échantillon vivaient en milieu rural (Village Kawama) soit une plus grande atteinte par rapport aux milieux urbains. D’autres auteurs sur le continent ont fait le même constat que nous. Par exemple, Arama 2009 [14] a trouvé, dans son étude portant sur les aspects épidémiologiques de la malnutrition infanto-juvénile dans le district de Koutiala au Mali que la proportion d’enfants malnutris est plus importante en milieu rural que dans les centres urbains. Globalement il avait noté une différence de prévalence de l’ordre de 20% entre le monde rural et le milieu urbain. La même situation est décrite par Boubacar, qui dans son étude trouvait que tous les malades de son échantillon étaient issus de couche socio-économique faible. Les parents étaient en général de petits employés ou des ouvriers [15].

On serait tenté d’expliquer ce fait par le bas niveau socio-économique et le faible niveau d’instruction en général des populations vivant dans cette contrée. Le niveau socio-économique du ménage est lié à la malnutrition sous toutes ses formes. En effet un bas niveau socio-économique du ménage expose tout enfant qui en est issu à la malnutrition. Cette relation a été trouvée dans les études menées par Sinnaeve O 2006 [12]. Les effets induits d’un niveau socio-économique précaire influencent la qualité, la quantité et la diversité des aliments consommés au sein de la famille mais aussi l’hygiène du cadre de vie et la survenue des maladies, toutes choses favorisant la survenue d’un état de malnutrition. Cependant, pour Arama 2009 [14], la profession du père, le niveau d’instruction du père et de la mère, la taille de la famille et le rang dans la fratrie ne sont pas des facteurs déterminants de la malnutrition. La profession du père et le niveau d’instruction des parents qui semblent être des baromètres du niveau socio-économique ne sont pas liés à la survenue de la malnutrition. Cette situation trouverait, selon lui, son explication au niveau de l’échantillonnage. En effet, les différents facteurs pris isolement semblent moins discriminants que l’indice composite qui semble mieux refléter le niveau de vie des familles [14]. Dans notre étude, le niveau socioéconomique bas contraste toutefois avec le taux assez élevés d’instruction de ces populations puisque 47,2% des mères des enfants de notre échantillon affirmaient avoir un niveau d’études secondaires.

Le mode d’alimentation des enfants est associé à la malnutrition, une différence est observée entre les enfants sous allaitement maternel exclusif et ceux qui sont sous régime familial. Le régime familial semble insuffisant ou tout au moins inadapté aux enfants récemment sevrés. Dans notre étude, 51% d’enfants dans la tranche d’âge entre 23 et 59 mois bénéficiaient d’un régime composé des mets familiaux. 0,6% de femmes ont suivi les recommandations de l’ANAES 2002 [20] et ont nourri exclusivement leurs enfants au lait maternel dans la tranche d’âge de 0 à 6 mois. Ce taux d’allaitement exclusif est inférieur à celui noté à l’échelle mondial qui était de 40% en 2012, mais également inférieur à la moyenne nationale qui est de 36% en RDC, selon l’Enquête démographique et santé (EDS) en septembre 2008, idem en 2012 [21]. Ce taux est aussi inférieur à celui du Ghana: 53%. Par contre Asfaw et al 2015 [22] en Ethiopie. Ont enregistrés au total, 90,5% des nourrissons qui ont été nourris exclusivement au sein pendant quatre à six mois, ce taux faible enregistré dans notre étude et ailleurs fait suite au manque de sensibilisation et d’information [23].

### Données anthropométriques des enfants malnutris

Les différents types de malnutrition par carence sont représentés dans notre échantillon. Ainsi, au cours de notre étude, la malnutrition chronique a été la plus représentée puisque 85% des enfants de notre échantillon présentaient un retard de croissance par rapport à leur âge contre 15,5% d’enfants qui souffraient de malnutrition aigüe. Parmi ceux-ci, respectivement 62,8% avaient présentés un tableau de malnutrition chronique sévère, 22,2% de malnutrition chronique modérée; 4,4% souffraient de malnutrition aigüe sévère et 11,1% de malnutrition aigüe modérée. Signalons en outre que 70% d’enfants de notre étude étaient en insuffisance pondérale de modérée (37,2%) à sévère (32,8%). Nos résultats sont différents de ceux de Boubacar 2009 [15] qui, dans son étude, rapporte 107 cas de malnutrition aigüe sévère, soit 70% de son échantillon et 6,1% de malnutrition aigüe modérée; également différents de ceux de Arama 2009 [15] qui avait trouvé que l’insuffisance pondérale était la plus importante puisque plus de 43,6% d’enfants de son échantillon en souffraient. Elle sévissait sous sa forme sévère dans plus de quatre cas sur dix. La malnutrition chronique était, dans son étude, présente chez 38,7% des enfants. Elle se manifestait sous sa forme sévère chez près de 18 enfants sur 100. Quant à l’émaciation (Poids/taille), elle concernait plus de 20% des enfants âgés de zéro à cinq ans. Plus de trois enfants sur 10 émaciés étaient atteints par la forme sévère. Tandis que Asfaw et al, 2015 [22] trouve une Prévalence du retard de croissance, l'insuffisance pondérale et l'émaciation chez les participants à l'étude étaient de 47,6%, 29,1% et 13,4% respectivement. Prévalence du retard de croissance sévère, d'insuffisance pondérale et l'émaciation chez les enfants étaient de 20,2%, 6% et 3,9% respectivement. Ces taux sont inférieurs à ceux observés dans notre étude.

Selon la gravité de la malnutrition, nous nous sommes basés sur la classification de Kanawati et al 1970 [24] qui tient compte du rapport périmètre brachial sur périmètre crânien. Notre étude révèle que 51,1% des enfants présentaient une malnutrition modérée, 25,6% était sévèrement malnutris et 23,3% légèrement malnutris.

### Les signes cliniques les plus rencontrés au cours de la malnutrition

Nous avons constaté dans notre étude, que le tableau clinique des enfants malnutris était dominé par la toux et ou pneumopathie dans 42,50%, la gastroentérite dans 38,55%, les dermatoses ont présenté 22,91% de cas, 22,35% ont présenté la fièvre, 19,0%, enfants ont présenté les œdèmes, 8,38% d’enfants ont présenté la pâleur, enfin hépatomégalie 1,68% et splénomégalie à 2,89% sont les signes les moins fréquemment observés. Tandis que les biens nourris ou les enfants en bonne état nutritionnel ont présenté plus les splénomégalies, les fièvres et les verminoses.

Concernant toujours la clinique, 36,7% ont souffert d’une entérite; Les affections associées à la malnutrition restent les mêmes mais avec une répartition diversement rapportée dans la littérature. Ainsi, Zebib HS 1984 [25], dans une étude faite sur la malnutrition proteino-énergétique, a décrit la diarrhée comme la première association morbide à la malnutrition proteino- énergétique dans des proportions pratiquement égale à nos résultats, soit 36,01% des cas observés. Boubacar 2009 [15] rapporte quant à lui une répartition légèrement inférieure à la nôtre, soit une association malnutrition aigüe, et la diarrhée représentent 23,1%. La diarrhée étant fréquemment rencontrée dans la malnutrition aigüe, du fait des parasitoses, des infections et de la malabsorption. Notre fréquence représente presque la moitié du constat qui a été fait par Ouédraogo et al 2012 [26] qui retrouve la diarrhée dans 60,9% des cas, Koum DK et al 2014 [27] a trouvé que principales complications étaient les gastroentérites (45,71%). Asfaw et al 2015 [22] trouve 48,7% et 25,1% des enfants avaient la diarrhée dans le 12 derniers mois et 2 semaines avant la collecte de données respectivement. Ainsi, nous pouvons dire que la diarrhée au cours de la malnutrition chez les enfants est très fréquente.

Nous avons enregistrés 42,5% de pneumopathies contrairement à Boubacar 2009, Ouédraogo et al 2012 et Banapurmathc 1994 [15, 26, 28] qui ont trouvé respectivement 24,4%, 19,8% et 31,8% justifiant ce taux par le fait que la malnutrition pourrait être responsable d’une défaillance du système immunitaire du sujet, ce qui pourrait le rendre vulnérable a des nombreuses infections.

Nous avons enregistrés 22,35% de cas de fièvre alors que Ouédraogo et al; 2012 [26] a trouvé que 71,3% d’enfant avaient la fièvre. L’hépatomégalie était rare dans notre étude soit 1,68% des signes, contrairement à Ouédraogo et al 2012 [26] qui a eu 14,6% d’hépatomégalie. Shindano, 2006 [29] a trouvé l’hépatomégalie chez 14,5% de sa population des malnutris avec œdème et 0% dans le marasme. Au Cameroun en 1983 Pondi [30] a trouvé l’hépatomégalie chez 25/34 (70%) des enfants présentant les œdèmes et chez 7/26 (22%) chez les enfants sans œdèmes. L’hépatomégalie semble plus fréquente chez Pondi, mais il existe un problème sur l’échantillon; la taille était 6 fois moins que notre échantillon.

Concernant les œdèmes, nous les avons enregistrés que dans 19% et la majorité de nos enfants étaient sans œdèmes. Le même constat a été fait par Ouédraogo et al 2012 [26] ont noté une fréquence plus élevée des MAS sans œdèmes (77,97%) comparativement aux MAS avec œdèmes (22,03%). Ceci est conforme aux données acquises sur la malnutrition et la plupart des études retrouvent cette prépondérance de la MAS sans œdèmes mais à des proportions différentes: De Lange en 2010 en Afrique du Sud (66,7%) [31], Bichet 2008 [32] en Somalie ont trouvé la malnutrition sans œdèmes dans 71% et Sedgho, 2009 [33] au Burkina Faso a trouvé 80% de malnutris sans œdèmes. Par contre Rytter MJ et al 2015 [34] a ont trouvé une fréquence plus élevée de la MAS avec œdèmes (Sur les 120 enfants inclus, 77 (64%) présentaient une malnutrition avec œdème. Les enfants malnutris Œdémateux étaient légèrement plus âgés (17,7 vs 15,0 mois, p = 0,006), alors que Bitwe et al 2006 [35] au Cameroun rapporte dans sa série 46,2% d’enfants avec œdème.

Le mécanisme d’apparition des œdèmes au cours de la malnutrition reste encore peu élucidé et la répartition des différentes formes n’a pas encore une explication qui fasse le consensus.

## Conclusion

La clinique de l'enfant malnutri est dominée par un déficit staturo - pondéral avec le retard de croissance ou une malnutrition chronique présente chez 85% d'enfants; soit 22,2% de forme modéré et 62,8% de forme sévère. La toux et ou pneumopathie (42,50%), la diarrhée et les vomissements ou la gastroentérite (38,55), les lésions dermatologiques ou dermatoses (22,91%), et la fièvre (22,35%) les œdèmes (19,0%). La pâleur, Hépatomégalie et splénomégalie sont des signes les moins fréquemment observés.

### Etat des connaissances actuelle sur le sujet

- La malnutrition est un problème de santé publique dans les pays en voie de développement. La RD Congo notamment est l'un des pays les plus concernés;
- Dans sa forme aigue, elle se présente sous plusieurs tableaux, dont les plus connues sont le marasme et le kwashiorkor, qui constituent des défis importants par rapport à la prise en charge.

### Contribution de notre étude à la connaissance

- Elle permet de décrire clairement les signes cliniques les plus rencontrés dans la prise en charge des malnutris sévères, et par conséquent de déterminer les axes de prise en charge les plus importants dans notre milieu (aspects environnementaux: alimentation, écologie, etc).
